# Eye movement characteristics in male patients with deficit and non-deficit schizophrenia and their relationships with psychiatric symptoms and cognitive function

**DOI:** 10.1186/s12868-021-00673-w

**Published:** 2021-11-24

**Authors:** Lin Zhang, Xiangrong Zhang, Xinyu Fang, Chao Zhou, Lu Wen, Xinming Pan, Fuquan Zhang, Jiu Chen

**Affiliations:** 1 Department of Geriatric Psychiatry, The Affiliated Nanjing Brain Hospital of Nanjing Medical University, No. 264 Guangzhou Road, Nanjing, 210029 Jiangsu China; 2 Department of Psychiatry, The Second People’s Hospital of Jiangning District, No. 50 ChenLing Road, Nanjing, 210003 Jiangsu China; 3 Institute of Neuropsychiatry, The Affiliated Brain Hospital of Nanjing Medical University, No. 264 Guangzhou Road, Nanjing, 210029 Jiangsu China

**Keywords:** Deficit schizophrenia, Exploratory eye movement, Mattis Dementia Rating Scale, Cognitive function

## Abstract

**Background:**

The cognitive impairment pattern of deficit schizophrenia (DS) is centered on an impaired attention function. Previous studies have suggested that the exploratory eye movement (EEM) tests reflect attention deficits in patients with schizophrenia. However, no study has investigated the characteristics of eye movement in DS in the Chinese Han population. This study aimed to investigate the pattern of eye movement characteristics in DS patients and to examine whether eye movement characteristic is associated with serious negative symptoms and cognitive decline in this schizophrenia subtype.

**Methods:**

A total of 86 male patients [37 DS and 49 non-deficit schizophrenia (NDS)] and 80 healthy controls (HC) participated in this study. Clinical symptoms were assessed using the Scale for the Assessment of Positive Symptoms (SAPS) and Scale for the Assessment of Negative Symptoms (SANS). Cognitive function was assessed using the Mattis Dementia Rating Scale (MDRS-2). Eye movement data of subjects were collected using an eye movement tracking analyzer.

**Results:**

There were significant differences in the overall eye movement data and cognitive test scores among the three groups (all *P* < 0.001). Both DS and NDS schizophrenia subgroups showed more severe eye movement and cognitive impairment compared with the control group. The number of eye fixations (NEF), total of eye scanning length (TESL), and cognitive function in DS patients were significantly lower than those in NDS patients. The discriminant analysis (D score) was higher than that of the control group (*P* < 0.001). In the DS group, the inattention factor of SANS was negatively correlated with the attention factor (r = − 0.545, *P* = 0.001) and structure factor of cognitive (r = − 0.389, *P* = 0.023), the affective flattening factor of SANS was negatively correlated with TESL (r = − 0.353, *P* = 0.041) and initiation/retention factor of cognitive (r = − 0.376,*P* = 0.028). TESL was found to positively correlate with the MDRS-2 total score (r = 0.427, P = 0.012), attention factor (r = 0.354, P = 0.040), and memory factor (r = 0.349, P = 0.043) in the DS group, whereas the mean of eye scanning length (MESL) positively correlated with cognitive impairments in the NDS group. The negative symptoms showed no significant correlation with cognition in the NDS group.

**Conclusions:**

Total of eye scanning length may be a characteristic eye movement symptom in DS patients, which is associated with serious negative symptoms and cognitive impairment in this schizophrenia subtype.

**Supplementary Information:**

The online version contains supplementary material available at 10.1186/s12868-021-00673-w.

## Introduction

Schizophrenia is a common and severe mental disease in clinical practice, which leads to social dysfunction [1], and might be the most disabling of all mental disorders [2]. Schizophrenia ranked 12th among 310 diseases and injuries worldwide in 2016 [3]. Charlson et al. reported the Global Burden of Disease (GDB) estimates for prevalence and disease burden of schizophrenia for all countries in 2016, and founded that about 21 million people worldwide suffer from schizophrenia, a number that will continue to rise as populations age and grow. Significant population growth and ageing have led to a large and increasing burden of disease caused by schizophrenia, particularly in middle-income countries [4]. The treatment of schizophrenia has made considerable progress, but about one-third of patients still have an unfavorable prognosis and continue to show decline in social functioning [5]. In 1988, Carpenter et al. proposed the concept of Deficit Schizophrenia (DS) subtype [6], characterized by primary and persistent negative symptoms; this classification is stable, with few changes in its core symptoms. However, the cognitive impairment pattern in DS is centered on the impaired attention function [7]. In addition, DS patients are characterized by familial aggregation, high birth rate in summer, poor long-term clinical efficacy, and early decline in social function [8, 9].

In 1972, Moriya et al. first reported eye movement abnormalities in schizophrenia patients using the exploratory eye movement (EEM) test [10]. Since then, most studies have consistently reported EEM disorders in schizophrenia patients [11–20]. EEM examination includes observation of the number of eye fixations (NEF), responsive of search scores (RSS), total of eye scanning length (TESL), the mean of eye scanning length (MESL), and the discriminant analysis (D score) [13, 21, 22]. NEF and RSS have been reported to reflect some characteristics of the cognitive function, including cognition, memory, attention, and other mental states of individuals [11, 23]. EEM examination has been regarded as a biological indicator of schizophrenia [14], which can reflect visual cognitive dysfunction and attention deficit in patients with schizophrenia [24, 25]. Previous studies have shown that most patients with schizophrenia have abnormal eye trajectories [14], a smaller number of fixations on memory tasks, and a narrower range of eye movements compared with healthy controls [11, 19, 26–28]. Existing research has shown that TESL and RSS in patients with schizophrenia are negatively correlated with negative symptoms such as emotional withdrawal and flatness [14].

Ample studies have further reported that patients with DS have more severe impairment in almost all cognitive domains [29]. Especially executive function [30, 31] and speech fluency [32–36] compared with patients with non-DS (NDS). Réthelyi et al. found that neurocognitive performance in general and in all cognitive domains except short-term memory were significantly reduced in patients with schizophrenia with deficits. Patients with DS and NDS had greater problems with non-verbal flexibility (TMT) than with linguistic cognitive flexibility (VFT P) [36]. Tyburski et al. found that the concept formation level of DS patients was lower than that of NDS patients, and the non-verbal cognitive flexibility level of DS patients was lower than that of NDS patients [37]. Negative symptoms are a core component of DS [6], and are associated with a large proportion of long-term morbidity and poor functional outcome cases in DS patients.

At present, the etiology of schizophrenia is complex, involving multi-gene inheritance, the pathogenesis has not been clear, the diagnosis is based on clinical professional psychiatrists, through the understanding of the patient's history and mental examination, referring to clinical diagnostic criteria for diagnosis, lack of specific objective diagnostic indicators. In previous studies, the eye movement pattern of patients with schizophrenia was significantly different from that of normal or non-schizophrenic patients, and the sensitivity and specificity of EEM were greater than 70% and 80% in distinguishing schizophrenic patients from non-schizophrenic patients, respectively [8, 21, 38, 39]. In 2009, Suzuki and his team performed a series of deterministic analyses of SZ and non-SZ subjects using the EEM examination study alone, Of 251 patients with clinically diagnosed SZ, 184 were determined to be true positive (sensitivity 73.3%) [21]. These results suggest that EEM may contribute to the clinical diagnosis of schizophrenia. However, it is unclear whether there is a difference in eye movement data between patients with DS and patients with NDS.

Therefore, the purpose of the study was to compare exploratory eye movements of healthy controls with those of DS patients, NDS patients, the participants were also assessed by the Revised Mattis Dementia Rating Scale (MDRS-2) for measuring cognitive functions [40], such as sustained attention and memory function. To determine if there are any differences in eye movement and cognition between DS and NDS patients. The second aim is to assess which stimulus factors or parts of the stimulus factors are responsible for the significant differences. We hypothesized that patients with DS and NDS have different cognitive patterns and eye movement characteristics, and that eye movement and cognitive function have their own correlation characteristics in the two subtypes of schizophrenia.

## Methods

### Participants

In all, 166 male participants were recruited in this study, including 86 schizophrenia patients who were clinically stable (37 DS and 49 NDS) and 80 health control (HCs). The schizophrenia patients were recruited from the Department of Psychiatry at the Second People's Hospital of Jiangning District in Nanjing, Jiangsu Province, China. The following individuals were included in the analysis: (1) individuals diagnosed with schizophrenia according to Diagnostic and Statistical Manual of Mental Disorders, Fourth Edition (DSM-IV), confirmed by the Chinese version of the Structured Clinical Interview for DSM-IV (SCID-I) [41]; (2) male, right-handed, Chinese Han patients aged between 25 and 65 years; and (3) individuals showing stable psychiatric symptoms and taking antipsychotic medication for at least 12 months based on the medical record. The exclusion criteria included the following: neurological disorders, severe comorbid conditions, head trauma, mental retardation, alcoholism or substance abuse disorder, and a history of previous electroconvulsive therapy. All DS and NDS patients were diagnosed according to the Chinese version of the Schedule for the Deficit Syndrome (SDS) [42].

The 80 male HCs were recruited from the local community and matched for age and handedness of the enrolled patients. The inclusion criteria: (1) male, right-handed, Chinese Han patients aged between 25 and 65 years; (2) Years of education ≥ 6 years. (3) These healthy subjects were assessed using unstructured clinical interviews to obtain basic demographic information as well as psychosocial information (age, education, living and marital status, family background, etc.) to rule out individuals with organic brain disorders, mental retardation or severe head trauma, and a personal or family history of mental disorders. The exclusion criteria: None of them had any drug or alcohol abuse/dependence, nor did they have severe visual impairment or red-green color blindness. They had no history of mental disorders personally or in their families. The study was approved by the Institutional Ethical Committee for clinical research of the Second People's Hospital of Jiangning District in Nanjing, and written informed consent was obtained from all participants.

### Clinical and neuropsychological assessment

#### Clinical evaluation

After consistency training, a semi-structured interview that achieved a high inter-rater reliability [intraclass correlation coefficient (ICC) = 0.84] was conducted by two senior attending physicians. The deficit syndrome was determined according to the Chinese version of SDS [43]. The diagnostic criteria for SDS include the presence of two or more of the following negative symptoms: a deficit syndrome that has reached clinical significance, has persisted for more than 12 months, and has persisted during periods of clinical stability. These symptoms were primary or idiopathic and not secondary to depression, anxiety, drug side effects, psychotic symptoms, or mental retardation. Our diagnostic criteria were in line with the DSM-IV diagnosis of schizophrenia. The Scale for the Assessment of Positive Symptoms (SAPS) and the Scale for the Assessment of Negative Symptoms (SANS) was used to evaluate the positive and negative symptoms of patients, respectively, for assessing the severity of schizophrenia symptoms. SANS scale was classified into separate categories including diminished expression, social amotivation, and inattention factors based on the findings of the most comprehensive factor analysis of the 19-item SANS to date [44].

#### Neurocognitive assessments

Each participant was assessed using MDRS-2, based on previous reports on cognitive process assessment for each task. These cognitive measures were further divided into five areas of reasonable motivation: continuous vigilance/attention (hereinafter referred to as continuous attention), initiation/retention, concept formation, structure, and memory [40].

### Exploratory Eye Movement (EEM) recording

The EEM examination was performed in 86 patients with schizophrenia and 80 HCs using DEM-2000 eye movement detection system (Shanghai Dikang, China). The EEM test procedure was based on that reported by Kojima et al. [12]. A standard 9-point calibration was performed before task initiation. A monocular sampling method was used to track the pupil of the dominant eye. EEM images were recorded on a videotape with an eye-mark recorder based on the reflection of infrared light on the cornea. The numbers of eye fixations (NEF), responsive of search scores (RSS), total eye scanning length (TESL), and mean eye scanning length (MESL) of the patient during the first 15 s of viewing a target figure was analyzed [14, 21, 22].

#### Eye movement recording procedure

First, the subject was comfortably seated in the viewing room, an eye camera that detected corneal reflection of infrared light to identify eye movements, and an LCD monitor that displayed target figures for EEM tasks were included in this system. Then, three horizontal S-shaped figures were projected onto a screen positioned at 30 cm directly in front of the subject’s eyes. The first S-shaped figure (S1) was displayed on the screen for 15 s. The second and third S-shaped figures (S2, S3), which were slightly different from the first one, were then displayed on the screen, each figure lasting for 15 s. Following this, the subject was asked whether the last two figures differed from the first figure and, if they did, how did they differ.

The gaze point (NEF) is the total number of points in the eye fixation pattern S1 within 15 s, and the duration of a certain point in the eye fixation image should be more than 200 ms. The RSS score is calculated by dividing S2 or S3 into 7 areas. The instrument measures the number of areas that the eyes focus on, for a total of 5 s. As long as the subjects' eyes gaze at a certain area, one point is counted, regardless of how many times this area is looked at. Therefore, the maximum RSS score for each image is 7 points, and the total RSS score for S2 and S3 is 14 points. The D score is calculated according to the discriminant analysis formula, D = 10.265 − [(0.065 × NEF) + (0.871 × RSS)]. The extent of the eye fixation point is TESL. MESL is the mean extent of eye movement. In the digital eye-mark recording system, the detected eye movements were automatically analyzed by a digital computerized EEM analyzer [14, 21].

### Statistical analysis

All statistical analyses were performed using the SPSS 26.0 software. We have used Kolmogorov–Smirnov statistical method to check the normal distribution of variables. The continuous variables of demographics, clinical symptoms, eye movement, and cognitive function between groups are presented as mean ± SD or Median (quartile spacing), and using two sample t-tests, analysis of variance (ANOVA) or Non-parametric test for comparisons, as appropriate. The qualitative data were analyzed by Chi-square test. EEMs, cognitive function, and psychiatric symptoms were compared using two sample *t*-tests between DS and NDS groups. Age and years of education were used as covariables to conduct covariance analysis on the data of the two groups. *P* < 0.05 was considered statistically significant.

We have used Kolmogorov–Smirnov statistical method to check the normal distribution of variables. To determine the group differences in eye movement and cognition function, statistical comparisons were performed on eye movement indices (such as NEF, RSS, D score, TESL, and MESL) and cognitive function indices (such as continuous vigilance/attention, initiation/retention, concept formation, structure, and memory) among the three groups Non-parametric tests. Partial correlation analysis between eye movements and neurocognitive domain and clinical variables (with age, years of education, and duration of disease as covariate) was conducted in the two patient groups. The significance level was set at *P* < 0.05. Bonferroni at *P* < 0.05 was applied to correct for multiple comparisons. After comparing the differences in various indicators between the two subgroups, the variables with differences (*P* < 0.05) between the two subgroups were finally included (the duration of disease, NEF, TESL, concept formation, structure and memory) in the stepwise logistical regression analysis to explore the independent risk factor for DS. The G*Power 3.1.9.2 program (http://www.softpedia.com/get/Science-CAD/G-Power.shtml) was used to run a power calculation and determine the effect size [45].

## Results

### Demographic and clinical characteristics

Demographic and clinical characteristics of the subjects are presented in (Table 1). The analysis of variance (ANOVA) results showed significant differences in age (*F* = 9.819, *P* < 0.001) and education (*F* = 28.024, *P* < 0.001) among the three groups. Bonferroni’s post-hoc comparisons revealed shorter education periods in the DS (*P* < 0.001) and NDS (*P* < 0.001) groups relative to those in the HC group, although the two patient subgroups did not differ significantly (*P* = 0.969). The DS (*P* = 0.001) and NDS (*P* = 0.009) patients were older than the HC subjects, but there was no significant difference between the two patient subgroups (*P* = 0.093). Our results indicated that significant differences in family history (*P* < 0.001) and marital status (*P* < 0.001) among the three groups, but the two patient subgroups had no significant differences in terms of family history (*P* = 0.209), marital status (*P* = 0.754), the mean age at onset, smoking status, positive symptoms, and antipsychotic medicine dosage (chlorpromazine equivalents), except for duration of disease (*t* = 3.771, *P* < 0.001) in the DS group. The DS patients showed more severe negative symptoms (*P* < 0.001) than the NDS patients.

**Table 1:** Demographics and clinical characteristics for DS, NDS and HC groups

	DS (n = 37)	NDS (n = 49)	HC (n = 80)	*F/Z*/χ^2^/*t*	*P*
Age (years)	50.00 ± 10.509^△^	45.86 ± 10.809^#^	40.48 ± 11.842	9.819	< 0.001
Education (years)	9.65 ± 2.251^△^	10.35 ± 3.244^#^	13.78 ± 3.590	28.024	< 0.001
Age at onset (years)	23.7365 ± 6.76	26.61 ± 9.808		− 1.612	0.111
Duration of disease (years)	26.27 ± 9.137^**^	19.04 ± 8.541		3.771	< 0.001
CPZ-equivalent daily dose (mg/day)	375 (280)	360 (210)		-0.725	0.468
Marital status [example (%)				60.169	< 0.001
Unmarried	30 (33.7)	41 (46.1)	18 (20.2)		
Married	7 (9.1)	8 (10.4)	62 (80.5)		
Family history [example (%)]				40.653	< 0.001
Positive	17 (51.5)	16 (48.5)	0 (0.0)		
Negative	20 (15.0)	33 (24.8)	80 (60.2)		
Use rate of anticholinergic drugs [example (%)	17 (54.8)	14 (45.2)		2.761	0.097
Smoking ratio (%)	13 (18.6)	26 (37.1)	31 (44.3)	3.518	0.172
SDS total score	13.62 ± 1.846^**^	8.86 ± 2.111		10.927	< 0.001
SAPS total score	9.22 ± 2.083	9.67 ± 0.944		− 1.364	0.176
Hallucinations	1.57 ± 0.603	1.57 ± 0.500		− 0.259	0.796
Delusions	2.08 ± 0.894	2.29 ± 0.540		− 1.317	0.191
Bizarre behavior	3.05 ± 0.911	2.98 ± 0.989		0.357	0.722
Positive thought disorder	2.54 ± 0.869	2.84 ± 0.624		− 1.840	0.069
SANS total score	44.81 ± 5.929^**^	31.27 ± 4.729		11.786	< 0.001
Affective flattening	11.30 ± 1.525^**^	9.12 ± 1.889		5.904	< 0.001
Alogia	10.76 ± 2.006^**^	6.90 ± 1.403		10.000	< 0.001
Avolition	8.65 ± 1.495^**^	5.53 ± 1.101		10.687	< 0.001
Anhedonia	9.81 ± 2.526^**^	6.24 ± 1.164		7.972	< 0.001
Inattention	4.41 ± 0.927^**^	3.47 ± 0.68		5.180	< 0.001

Mean ± SD; Median (quartile spacing); DS: defificit schizophrenia; NDS: non-defificit schizophrenia; HC: healthy controls; SANS: the Scale for the Assessment of Negative Symptoms; SAPS: the Scale for the Assessment of Positive Symptoms; SDS: schedule for the defificit syndrome; CPZ: chlorpromazine

^**^P < 0.001 DS vs. NDS;

^*^P < 0.05 DS vs. NDS;

^△^*P* < 0.05 DS vs. HC;

^#^*P* < 0.05 NDS vs. HC

### Cognitive characteristics

The results of normality test showed that MDRS-2 score and subscale score among the three groups were all almost non-normal (P < 0.05). Then, we have conducted a Kruskal–Wallis non-parametric test. Our results showed significant differences among the three groups (all *P* < 0.001). Bonferroni’s post-hoc comparisons confirmed that both the DS and NDS patients performed worse than the HC subjects in each of the neuropsychological tests (all *P* < 0.001). Patients with DS, as compared with those with NDS, had significantly more severe impairment in most of the neuropsychological measures (Bonferroni corrected *P* < 0.05) (Table 2). As the family history and marital status varied among the HC subjects, DS and NDS patients, we further made a stratification by the family history and marital status to compare the cognitive function among these three groups, the results showed in the Additional file 1: Table S1 and Table S2.

**Table 2:** Eye movement and cognitive function for DS, NDS and HC groups

	DS (n = 37)	NDS (n = 49)	HC (n = 80)	H	P	DS VS NDS		DS VS HC		NDS VS HC	
						H	P	H	P	H	P
NEF(score)	22 (14)	27 (11)	30 (4)	44.41	< 0.001	− 2.480	0.013	− 6.381	< 0.001	− 4.015	< 0.001
RSS (score)	3 (2)	4 (2)	7 (1)	96.59	< 0.001	− 1.246	0.213	− 8.394	< 0.001	− 7.704	< 0.001
D score	5.9 (2.3)	4.9 (2.8)	2 (1.3)	98.26	< 0.001	1.424	0.154	8.553	< 0.001	7.664	< 0.001
TESL	120.2 (123)	192.6 (155)	281.8 (112)	60.06	< 0.001	− 2.706	0.007	− 7.636	< 0.001	− 4.820	< 0.001
MESL	6.3 (4.9)	8.9 (4.8)	11 (5.6)	40.06	< 0.001	− 1.156	0.248	− 5.583	< 0.001	− 4.731	< 0.001
MDRS-2 score	82 (34.5)	112 (26)	139 (6)	124.92	< 0.001	− 2.577	0.010	− 10.105	< 0.001	− 7.981	< 0.001
Continuous vigilance/ attention	34 (5.5)	36 (2)	37 (0)	80.50	< 0.001	− 1.888	0.059	− 8.031	< 0.001	− 6.535	< 0.001
Initiation/ retention	13 (11)	23 (13)	36 (2)	121.25	< 0.001	− 2.316	0.021	− 9.856	< 0.001	− 8.022	< 0.001
Concept formation	17 (18)	28 (9.5)	35 (3)	108.45	< 0.001	− 2.606	0.009	− 9.504	< 0.001	− 7.287	< 0.001
Structure	5 (3.5)	6 (1)	6 (0)	49.01	< 0.001	− 2.913	0.004	− 6.792	< 0.001	− 3.946	< 0.001
Memory	14 (9.5)	20 (5.5)	25 (0)	125.08	< 0.001	− 3.044	0.002	− 10.308	< 0.001	− 7.642	< 0.001

Median (quartile spacing); H: The effect size of nonparametric tests; P: significance level; NEF: the numbers of eye fixations; RSS: responsive of search scores; TESL: total eye scanning length; MESL: mean eye scanning length; MDRS: Dementia Rating Scale

### Characteristics of eye movement parameters

**Fig. 1:**
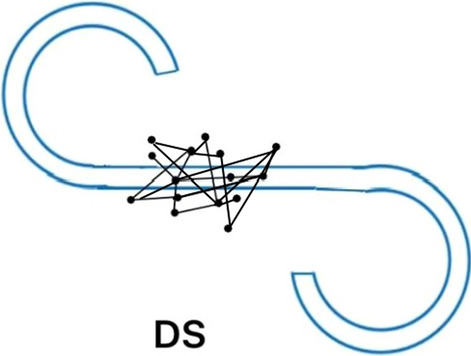
NEF: 15. TESL: 80.17 mm. MESL: 7.93 mm

**Fig. 2:**
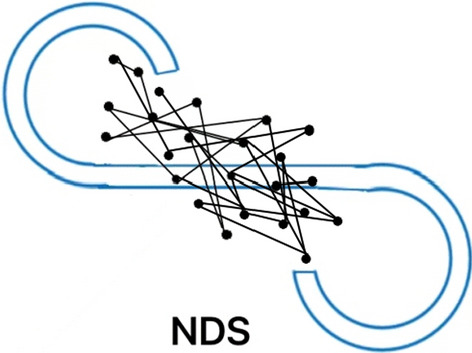
NEF: 24. TESL: 195.20 mm. MESL: 8.59 mm

**Fig. 3:**
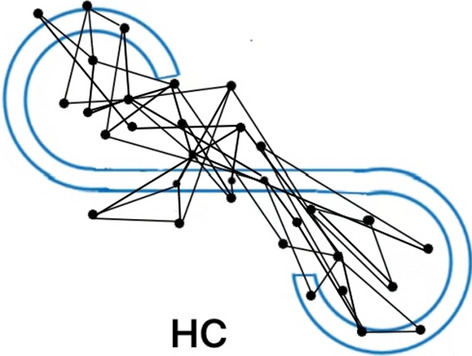
NEF: 32. TESL: 283.40 mm. MESL: 12.06 mm

Figures 1, 2, and 3 show a typical example of an eye scanning trajectory of a DS patient, an NDS patient, and a healthy control. The frequency of eye fixation points (NEF) were lowest and the total eye scanning length (TESL) were the shortest in DS patients. Non-parametric test analysis showed that there were statistically significant differences in eye movement indices among the three groups (all *P* < 0.001). As the family history and marital status varied among the HC subjects, DS and NDS patients, we further made a stratification by the family history and marital status to compare the eye movement parameters among these three groups, the results showed in the Additional file 1: Table S1 and Table S2. Bonferroni’s post-hoc comparisons showed that patients with DS (*P* < 0.001) and NDS (*P* < 0.001) had worse eye movement parameters than HC subjects. NEF and TESL in the DS group were significantly lower than those in the NDS group (Bonferroni corrected *P* < 0.05). There were no statistically significant differences in RSS, MESL and D value between the two groups (both *P* > 0.05) (Table 2, Figs. 1, 2, 3). The statistical power of our study to detect the difference of NEF, RSS, D score, TESL, MESL, MDRS-2 score, continuous vigilance/attention, initiation/retention, concept formation, structure, memory between patients and healthy controls were all 100%.

### Relationships between eye movement, clinical features, and cognitive domains

With age, years of education, and duration of disease as covariables, the clinical symptoms, eye movement indices, and cognitive indices in the DS and NDS groups were analyzed by partial correlation. The bizarre behavior factor of SAPS in the DS group was negatively correlated with NEF (r = − 0.545, *P* = 0.001) and memory factor (r = − 0.347, *P* = 0.045) and positively correlated with D value (r = 0.396, *P* = 0.020). The affective flattening factor of SANS was negatively correlated with TESL (r = − 0.353, *P* = 0.041) and initiation/retention factor of cognitive (r = − 0.376,*P* = 0.028). The inattention factor of SANS was negatively correlated with the MDRS-2 total score (r = − 0.468, *P* = 0.005), attention factor (r = − 0.545, *P* = 0.001), and structure factor (r = − 0.389, *P* = 0.023). TESL was positively correlated with MDRS-2 score (r = 0.427, *P* = 0.012), attention factor (r = 0.354, *P* = 0.040), and memory factor (r = 0.349, *P* = 0.043).

MESL was positively correlated with the inattention factor of SANS(r = 0.337, *P* = 0.022), MDRS-2 total score (r = 0.401, *P* = 0.006), initiation/retention factor (r = 0.349, *P* = 0.018), and concept formation factor (r = 0.392, *P* = 0.007) in the NDS group. The negative symptoms showed no significant correlation with cognition in the NDS group (Table 3, 4). Stepwise logistic regression analysis showed that duration of disease (odds ratio [OR] 1.081, 95% confidence interval [CI] 1.016–1.150, P < 0.05), memory (odds ratio [OR] 0.803, 95% CI 0.718–0.897, P < 0.001), and TESL (odds ratio [OR] 0.992, 95% CI 0.986–0.998, P < 0.05) were independently associated with DS.

**Table 3:** Correlation of eye movement with cognitive and clinical symptoms in DS group

		NEF	TESL	MDRS-2 total score	Continuous vigilance/attention	Initiation/retention	Structure	Memory
SAPS	Bizarre behavior	− 0.545**	− 0.226	− 0.210	− 0.009	− 0.137	− 0.019	− 0.347*
SANS	Affective flattening	− 0.178	− 0.353*	− 0.145	0.014	− 0.376*	0.046	− 0.206
	Inattention	− 0.120	− 0.323	− 0.468**	− 0.545***	− 0.270	− 0.389*	− 0.263
	TESL	0.518**	–	0.427*	0.354*	0.293	0.214	0.349*

*P < 0.05 after correction of multiple testing

**P < 0.01 after correction of multiple testing

***P < 0.001 after correction of multiple testing

**Table 4:** Correlation of eye movement with cognitive and clinical symptoms in NDS group

		NEF	MESL	TESL	MDRS− 2 total score	Initiation/retention	Concept formation
SAPS	Bizarre behavior	0.068	− 0.263	− 0.229	0.203	0.038	0.238
SANS	Inattention	− 0.122	0.337*	0.308*	0.121	0.017	0.222
	MESL	− 0.252	–	0.649**	0.401**	0.349*	0.392**

*P < 0.05 after correction of multiple testing

**P < 0.01 after correction of multiple testing

## Discussion

To the best of our knowledge, this is the first study to investigate eye movement characteristics in patients with DS and their correlation with clinical symptoms and cognition. The main findings of our study were as follows: in the DS group and NDS group, eye movement index and cognitive function were generally low, and the impairment of eye movement index in DS group (i.e., lower NEF and TESL) was greater than that in the NDS group. DS patients showed greater impairments in all cognitive function measures than NDS patients [29]. Both schizophrenia subgroups performed worse than the control group in every area of eye movement measurement and cognition. In addition, NEF was significantly correlated with SAPS bizarre behavior factor in the DS group, whereas TESL was correlated with SANS affective flattening factor, cognitive MDRS total score, attention factor, and memory factor in the DS group. Further study found that TESL in DS group was related to emotional state of SANS, memory function of cognitive and disease duration. TESL and memory function of DS patients significantly affected the patient's condition. However, MESL was associated with inattention of SANS, executive function, and visuospatial ability of cognitive in the NDS group. The two patient groups had different patterns of core eye movement damage, which suggested that DS might be an independent subtype of schizophrenia, and TESL might be a biomarker for the diagnosis of DS.

NEF and RSS reflect cognitive function such as memory and active attention, whereas TESL is associated with emotional withdrawal, and blunted effect and emotional relationship disorder [27]. The previous studies have found the lower the NEF, the shorter the TESL and the narrower the range of eye movement in the patients with schizophrenia, suggesting that limited scanning length may be a characteristic of schizophrenia [14, 19, 28]. Less eye movement was believed to be a sign of impaired visual cognitive function [12, 14, 15, 22]. A significant reduction in the scan length may be because of lack of sustained attention [27]. In this study, both DS and NDS patients presented with lower NEF and shorter TESL, which was largely compatible with several previous studies, indicating decreased eye movement in schizophrenia [14, 19, 28]. In addition, in our study, the differences of NEF and TESL between DS patients and NDS patients in Figs. 1 and 2 were significant, and the MESL of DS did not change significantly, indicating that they were state independent. Kojima et al. [14] reported in 1990 that TESL depended on clinical status and was a status marker. These studies also suggest that MESL abnormalities are specific to patients with chronic schizophrenia and are an indicator of a chronic state. Ryu et al. observed that individuals with schizophrenia may stare aimlessly at a certain part of the picture rather than properly focusing their attention on that part of the picture [27]. Shakow reported that people with schizophrenia were unable to focus their attention on the external environment or move in a meaningful direction [46]. In terms of face recognition, Williams et al. reported that under more difficult task conditions, schizophrenia patients could not focus their eye gaze on characteristic areas; they thought that these patients could not form the general gestalt of the initial registration, followed by abnormal stress of serial processing. These studies found that patients with schizophrenia in a variety of eye movement task execution process of aberrations [47]. Restricted facial visual scanning may indicate social cognitive impairment or may be a marker of vulnerability associated with disease features [48, 49]. These findings indicate that abnormal eye movement may be a common feature in schizophrenia, regardless DS and NDS, but the decrease was more pronounced in DS patients. These gaze points in DS patients were few and also relatively fixed and limited. The eyes of DS patients tend to be fixated at a single point or move within a particularly limited range, which significantly shortens the total eyeball distance. This may be closely related to the disease characteristics of DS patients. In our study, it was found that DS patients were more indifferent to the surrounding environment, passive contact, more passive in eye movement examination and scale evaluation, less speech, stiff expression after hearing questions, less eye movement and shorter TESL. Persistent negative symptoms caused the patient to be unable to concentrate on a particular instruction. DS patients with emotional withdrawal, anhedonia, resulting in patients in interpersonal communication and social interaction on the obstacles.

Schizophrenia patients have cognitive impairments in attention, working memory, language learning, visual learning, social cognition, and other aspects [50, 51]. Previous studies have reported that EEM abnormalities in schizophrenia are associated with cognitive impairment [52–55]. Several studies have shown differences in neurocognitive function between patients with DS and NDS [56, 57]. Previous studies have found that DS patients had more severe impairment in almost all cognitive domains when compared with NDS patients [29]. A meta-analysis by Cohen et al. reported evidence of moderate widespread cognitive impairment in patients with defect syndrome [58]. Previous studies have found that schizophrenia patients with persistent negative symptoms showed more severe continuous attention impairment in related cognitive assessments [59–61]. A modified version of MDRS was used in this study [40, 62, 63]. The present study found that both schizophrenia subgroups performed worse compared with the control group in every cognitive area. These reflected the overall impairment of cognitive function in schizophrenia. Compared with NDS patients, DS patients had more severe impairments in all neurocognitive domains, and it was correlated with the attention factors of SANS: the more severe the negative symptoms, the worse the neurocognitive function. However, NDS patients performed between DS and healthy controls in all cognitive domains, negative symptoms were not associated with cognitive function in the NDS group, which is consistent with previous reports of more severe cognitive impairment in DS patients [7, 36, 58]. There were significant differences in the neurocognitive impairment patterns between the two groups, mainly because of the differences in attention and executive function between the two groups. The persistent attention deficit may be the core cognitive impairment module in DS patients [29].

The severity of negative symptoms stabilized over time in patients with DS and NDS, while the severity of symptoms increased in patients with NDS. Thus, the widespread cognitive impairment in patients with DS is consistent with the earlier view that the deficiency syndrome is biologically unique and has more features of impairment than in patients with NDS [64]. A 2017 meta-analysis showed that cognitive deficits were more severe in DS patients, and language fluency was identified as one of the more severely affected cognitive areas, as alogia was a typical feature of the disease in schizophrenia patients with persistent negative symptoms [65]. Other studies have reported impaired sustained attention [7, 36, 58–61] and visuospatial memory [42, 66] in DS patients. Patients with DS have been previously reported to have abnormal frontal and parietal lobe function. In recent years, a number of studies have shown that compared with female patients with schizophrenia, total brain volume, ventricule-brain ratio, frontal and temporal lobe decreases are more significant in male patients with schizophrenia. Meanwhile, sexual dimorphism has been found in geometric abnormalities of the white matter corpus callosum in patients with schizophrenia [67, 68]. Wheeler et al. found neuroimaging abnormalities in fronto-parietal function in DS patients [69]. Rowland and Voineskos, using motion diffusion tensor imaging studies, have identified reproducible impairment of white matter tracts in frontoparietal and frontotemporal circuits important for emotional processing emotional expression and social-emotional function [70, 71], all of which are characteristic of impaired patients with defective schizophrenia. Recent imaging studies have consistently found that patients with DS have reduced gray [72–75] and white matter volumes [74] in the temporal lobe region compared with patients with NDS. These findings regarding the structural abnormalities in specific brain regions in patients with different schizophrenia subtypes may explain the differences in core cognitive impairment modules between the DS and NDS groups.

Furthermore, the present study found the specific correlations of eye movement and clinical features with cognition in the DS group, which may represent a possible compensatory response to the functional deficit in DS patients. The present study found that the attention factors in the SANS in the DS group were significantly negatively correlated with the total MDRS score, attention factors, and structural factors, whereas the negative symptoms in the NDS group were not correlated with cognition. that TESL and RSS in patients with reported that TESL and RSS in patients with schizophrenia are negatively correlated with negative symptoms such as emotional withdrawal and flatness [10, 14, 27, 28, 53, 54]. However, there was no significant correlation between positive symptoms and TESL and RSS. In the present study, NEF was significantly negatively correlated with the bizarre behavior factor of SAPS. Further, TESL was found to be negatively correlated with affective flattening factor of SANS in the DS group, but not in the NDS group. TESL was also positively associated with the total MDRS score, attention factor, and memory factor. In NDS patients, MESL was correlated with attention deficit of SANS, executive function and visuospatial ability of cognitive function, while negative symptoms were not correlated with cognition. This indicates that the shortening of total fixation distance in DS patients is related to the severity of negative symptoms and cognitive decline. The more severe the negative symptoms, the shorter the total fixation distance and the worse the cognitive function.

Recent studies have shown that negative symptoms play a key role in mediating the neurocognitive and social cognitive functions in patients with schizophrenia and in the prognosis of their functional outcomes [76]. Visual cognitive dysfunction in patients with schizophrenia was closely associated with the decline in social function, social status, independent living, working ability, and interpersonal communication ability [77–80], which may be the restrictions that hinder the social and vocational rehabilitation of patients [81]. DS is a subtype of schizophrenia. The characteristic negative symptoms of this subgroup include restriction of emotion, reduced range of emotion, poor speech, inhibition of interest, poor sense of purpose, and reduced social motivation [43, 57]. Further, patients with DS show more persistent, prominent negative symptoms and are associated with neurocognitive and social cognitive impairments [9]. Tsunoda et al. reported that a number of eye movement parameters were negatively associated with negative symptoms, particularly lack of willpower and blandness or dullness of emotion [82]. These findings suggested that exploratory eye movements are biomarkers for negative symptoms such as emotional withdrawal, emotional dullness, and mannerisms [27]. This clinical correlation suggested that negative effects on eye movement reflect underlying brain dysfunction that leads to communication disorders in relation to the environment and interpersonal relationships [10, 12]. In addition, this study showed that MESL was positively correlated with total MDRS score, onset/retention factor, and concept formation factor in the NDS group. No correlation of TESL and negative symptoms with cognition was found in NDS patients, suggesting significant differences in eye movement characteristics between the two subgroups, which may result from the biological characteristics distinguishing DS from NDS.

To date, eye-tracking dysfunction remains a characteristic disorder limited to the risk of schizophrenia, although other factors have been reported to be abnormal in patients with schizophrenia [83, 84]. This relatively economical, safe and far-reaching eye-movement technique can well reflect the cognitive deficits of psychiatric patients. Therefore, the correlation between visual cognitive pattern and brain region structure in patients with mental disorders, especially schizophrenia, needs further study. At present, some studies have reported the relationship between eye movement characteristics and brain morphology and structure in patients with schizophrenia, providing valuable reference for cognitive function and social dysfunction in patients with schizophrenia [85–88], and also providing objective theoretical basis for clinical diagnosis and treatment [87–89]. Our preliminary findings suggest that cognitive and eye movement features may be important index in identifying the special type of schizophrenia patients with defective syndrome, and clinicians should pay more attention to cognitive evaluation and eye movement features in those patients. Considering the refractory nature of negative symptoms, more exploration should be implemented based on this subtype patients.

In the present study, we did find that both DS and NDS patients showed different eye movement features compared to healthy controls, the preliminary findings support that eye movement features may be a valuable bio-marks in the diagnosis of schizophrenia. TESL may be a characteristic eye movement symptom in DS patients, which is associated with serious negative symptoms and cognitive impairment in this schizophrenia subtype. However, as a cross-section study, we could not explore is role in predicting the prognosis in those patients. Hence, future studies are warranted to verify our findings and to explore its effects in predicting the prognosis in patients with schizophrenia, and future studies used more commonly used cognitive evaluation tools are also warranted to verify our findings.

## Limitations of the study

Our study has several limitations. First, all patients were receiving antipsychotics, and although the type and dosage of antipsychotics were consistent between the two groups of patients, their influence on the study results cannot be ruled out. Second, patients in the two subgroups had different disease courses and age. Although the duration of disease and age had been controlled as a covariable in the statistical analysis of this study, it cannot be ruled out that it may have a certain influence on cognition. Third, the cognitive assessment tool we used was the Mattis Dementia Rating Scale (MDRS), so our results may not be generalizable to studies based on other tools, more studies with different tools are needed in the future to validate our findings. Fourth, we did not assess and control for the effects of IQ on cognitive function. In addition, only male patients were included in the present study, which may preclude our conclusion to female patients with schizophrenia, and further study need to explore the gender difference in eye movement features in DS and NDS patients. The future studies ought to also consider outpatients, female patients, and larger sample sizes (Additional file 1).

## Conclusions

In summary, our findings provide empirical evidence for association between eye movement and cognitive function in patients with DS and NDS. TESL is significantly associated with negative symptoms and impaired cognitive function. Specifically, abnormalities between the two patient groups emphasize that visuoemotional and social functions were characteristically impaired in DS Patients. Patients with DS have persistent negative symptoms that lead to visual cognitive deficits in novel and complex problem-solving environments, and these deficits are partly related to cognitive indicators of persistent attention and executive dysfunction. These deficits begin to show with a reduced ability to scan complex visual environments effectively and eventually lead to environmental and social impairments. The present study suggests that deficit syndrome may be a specific subtype of schizophrenia.

## Supplementary Information

**Additional file 1: Table S1.** Comparison of eye movement and cognitive function and eye movement parameters among for DS, NDS and HC groups stratified by marital status. **Table S2.** Comparison of eye movement and cognitive function and eye movement parameters among for DS, NDS and HC groups stratified by family history.

## Data Availability

Data available on request from the corresponding author.

## Abbreviations

ANCOVA: Analysis of covariance
CPZ: Chlorpromazine
DS: Deficit schizophrenia
DSM-IV: Diagnostic and statistical manual-IV
EEM: Exploratory eye movement
GDB: Global Burden of Disease
MDRS-2: Mattis Dementia Rating Scale
MESL: Mean of eye scanning length
NDS: Non-deficit schizophrenia
NEF: Number of eye fixation
RSS: Responsive of search scores
SANS: Scale for the assessment of negative symptoms
SAPS: Scale for the assessment of positive symptoms
SCID: Structured clinical interview for DSM-IV
SDS: Schedule for the Deficit Syndrome
SPSS: Statistical package for social sciences
TESL: Total of eye scanning length
