# Supplementary material for: Eye movement characteristics in male patients with deficit and non-deficit schizophrenia and their relationships with psychiatric symptoms and cognitive function
Source: BMC Neurosci. 2021 Nov 24;22:70. doi: 10.1186/s12868-021-00673-w (PMC8613938; doi:10.1186/s12868-021-00673-w)
Supplement: Supplementary file 1 — Additional file 1: Table S1. Comparison of eye movement and cognitive function and eye movement parameters among for DS, NDS and HC groups stratified by marital status. Table S2. Comparison of eye movement and cognitive function and eye movement parameters among for DS, NDS and HC groups stratified by family history. [file 12868_2021_673_MOESM1_ESM.docx]

**Additional material**

**Table S1. Comparison of eye movement and cognitive function and eye movement parameters among for DS, NDS and HC groups stratified by marital status**

| unmarried married  DS(n=30) NDS(n=41) HC(n=18) P DS(n=7) NDS(n=8) HC(n=62) P | | | | | | | | |
| --- | --- | --- | --- | --- | --- | --- | --- | --- |
| NEF | 20.5(15.5) | 27(11) | 29(4.3) | <0.001 | 27(13) | 26.5(14.8) | 31(4) | 0.011 |
| RSS | 3(3) | 4(2) | 7(1.5) | <0.001 | 4(1) | 4(2.8) | 7.5(1) | 0.002 |
| D score | 6(3) | 5(3.1) | 2.4(1.7) | <0.001 | 5.2(1.7) | 5(1.7) | 1.5(1.4) | 0.007 |
| TESL | 128(132.4) | 214.3(135.3) | 263(100) | 0.008 | 120.2(99.3) | 90(65.2) | 286.8(120) | <0.001 |
| MESL | 7.5(4.6) | 9.2(4.8) | 10.5(4) | <0.001 | 5(2.5) | 4.3(3.1) | 11.7(5.8) | <0.001 |
| MDRS-2 | 82.5(35) | 114(25) | 140.5(3) | <0.001 | 81(37) | 98.5(26) | 138.5(6) | <0.001 |
| attention | 34(5.2) | 36(2) | 37(0) | <0.001 | 35(8) | 35(3.2) | 37(0) | <0.001 |
| initiation/  retention | 13(12) | 25(12.5) | 36(1) | <0.001 | 16(9) | 19(7.5) | 36(2) | <0.001 |
| concept formation | 16.5(18.2) | 29(8) | 36(2.3) | <0.001 | 17(12) | 19.5(12) | 35(4) | <0.001 |
| structure | 5.5(3) | 6(1) | 6(0) | 0.002 | 3(3) | 5(3) | 6(0) | <0.001 |
| memory | 14(10) | 20(4.5) | 25(0) | <0.001 | 13(7) | 20(5.5) | 25(0) | <0.001 |

Note: [Median](C:/Program%20Files%20(x86)/Youdao/Dict/8.9.9.0/resultui/html/index.html#/javascript:;) (quartile spacing); NEF: the numbers of eye fixations; RSS: responsive of search scores; TESL: total eye scanning length; MESL: mean eye scanning length; MDRS: Dementia Rating Scale.

**Table S2. Comparison of eye movement and cognitive function and eye movement parameters among for DS, NDS and HC groups stratified by family history**

| Family history No family history negative  DS(n=17) NDS(n=16) HC(n=0) P DS(n=20) NDS(n=33) HC(n=80) P | | | | | | | | |
| --- | --- | --- | --- | --- | --- | --- | --- | --- |
| NEF | 18(22.5) | 24(12.3) | 0 | 0.406 | 23.5(14.3) | 28(7) | 30(4) | <0.001 |
| RSS | 3(2.5) | 4.5(3) | 0 | 0.018 | 4(2) | 4(2.5) | 7(1) | <0.001 |
| D score | 6.5(3.2) | 4.6(3.4) | 0 | 0.052 | 5.2(2.4) | 5.2(2.9) | 2(1.3) | <0.001 |
| TESL | 114(116.8) | 198.8(124.4) | 0 | 0.031 | 138.1(150.6) | 192.6(166.8) | 281.8(111.7) | <0.001 |
| MESL | 6.1(6.7) | 9.4(5.7) | 0 | 0.160 | 6.6(3.8) | 8.4(4.4) | 11(5.6) | <0.001 |
| MDRS-2 | 84(26) | 118(18) | 0 | 0.005 | 80(37) | 108(26) | 139(6) | <0.001 |
| attention | 35(6) | 36(0.7) | 0 | 0.077 | 33.5(5.5) | 35(3) | 37(0) | <0.001 |
| initiation/  retention | 14(11) | 28(12.5) | 0 | 0.012 | 13(12.2) | 22(10.5) | 36(2) | <0.001 |
| concept formation | 17(17) | 28.5(7.5) | 0 | 0.004 | 15(19.2) | 27(12) | 35(3) | <0.001 |
| structure | 6(2.5) | 6(1) | 0 | 0.731 | 4(5.5) | 6(1) | 6(0) | <0.001 |
| memory | 14(8.5) | 20.5(8) | 0 | 0.011 | 12.5(9.5) | 20(5.5) | 25(0) | <0.001 |

Note: [Median](C:/Program%20Files%20(x86)/Youdao/Dict/8.9.9.0/resultui/html/index.html#/javascript:;) (quartile spacing); NEF: the numbers of eye fixations; RSS: responsive of search scores; TESL: total eye scanning length; MESL: mean eye scanning length; MDRS: Dementia Rating Scale.
